# Low-dose inoculation of *Escherichia coli* achieves robust vaginal colonization and results in ascending infection accompanied by severe uterine inflammation in mice

**DOI:** 10.1371/journal.pone.0219941

**Published:** 2019-07-22

**Authors:** Valerie P. O’Brien, Nicole M. Gilbert, Tania Lebratti, Kavita Agarwal, Lynne Foster, Haina Shin, Amanda L. Lewis

**Affiliations:** 1 Department of Molecular Microbiology, Washington University School of Medicine, St. Louis, MO, United States of America; 2 Center for Women’s Infectious Disease Research, Washington University School of Medicine, St. Louis, MO, United States of America; 3 Department of Obstetrics and Gynecology, Washington University School of Medicine, St. Louis, MO, United States of America; 4 Center for Reproductive Health Sciences, Washington University School of Medicine, St. Louis, MO, United States of America; 5 Department of Medicine and Division of Infectious Diseases, Washington University School of Medicine, St. Louis, MO, United States of America; The Research Institute at Nationwide Children's Hospital, UNITED STATES

## Abstract

*Escherichia coli* infection of the female reproductive tract is a significant cause of disease in humans and animals, but simple animal models are lacking. Here we report that vaginal inoculation of uropathogenic *E*. *coli* strains UTI89 and CFT073 in non-pregnant, estrogen-treated mice resulted in robust colonization of the vagina and uterine horns, whereas titers of the lab strain MG1655 were significantly lower. Non-estrogenized mice also became colonized, but there was more variation in titers. A dose of 10^4^ colony-forming units (CFU) UTI89 was sufficient to result in colonization in all estrogenized mice, and we also observed bacterial transfer between inoculated and uninoculated estrogenized cage mates. UTI89 infection led to inflammation and leukocyte infiltration into the uterine horns as evidenced by tissue histology. Flow cytometry experiments revealed that neutrophil, monocyte and eosinophil populations were significantly increased in infected uterine horns. This model is a simple way to study host-pathogen interactions in *E*. *coli* vaginal colonization and uterine infection. There are immediate implications for investigators studying urinary tract infection using mouse models, as few *E*. *coli* are required to achieve reproductive colonization, resulting in an additional, underappreciated mucosal reservoir.

## Introduction

Bacterial infections of the female reproductive tract (FRT) are common and can carry serious consequences. *Escherichia coli* is an opportunistic pathogen and frequent cause of human disease [1], including in the FRT [2]. Some women are vaginally colonized with *E*. *coli* in the apparent absence of symptoms, with colonization rates ranging from ~5% [3, 4] to as high as ~25% [5–7]. In some women, vaginal *E*. *coli* may trigger a painful inflammatory condition termed aerobic vaginitis [3]. As well, *E*. *coli* residing in the vagina may be displaced into the bladder to cause urinary tract infections (UTI). Women with a history of recurrent UTI are more likely than healthy controls to harbor *E*. *coli* at the vaginal introitus [5, 8], and after sexual intercourse (a common UTI trigger [9]), vaginal introital *E*. *coli* colonization increases [10].

Ascending *E*. *coli* infection from the vagina to the upper FRT (i.e. uterine infection) is also a significant cause of disease both in pregnant and non-pregnant women [11, 12]. During pregnancy, ascending *E*. *coli* infection can lead to a wide range of negative consequences for the mother and child, such as neonatal or maternal sepsis, miscarriage and pre-term birth [2, 13–15]. Strikingly, *E*. *coli* is considered the most common infectious organism associated with stillbirth [13]. However, little is known about *E*. *coli* uterine infection outside of the context of pregnancy.

Despite the many negative health consequences of *E*. *coli* FRT colonization, the pathogenic cascade of *E*. *coli* in this niche is not well understood and only a few studies have used animals models to examine this topic, for example in mice [16, 17] and monkeys [18]. We sought to develop a simple mouse model of ascending *E*. *coli* FRT colonization after vaginal inoculation with model *E*. *coli* strains, in order to better understand this infection and to characterize the host immune response. We found that uropathogenic *E*. *coli* could readily colonize the mouse vagina and did so especially well in mice treated with exogenous estrogen to halt the natural estrous cycle. *E*. *coli* inoculation also resulted in ascending infection of the uterine horns, where it elicited histological inflammation and immune cell infiltration. In contrast, a lab strain of *E*. *coli* was highly attenuated in the FRT.

## Methods

### Ethics statement

All mouse experiments were conducted per the National Institutes of Health guidelines for the housing and care of laboratory animals. All experiments were performed in accordance with institutional regulations after review and approval by the Institutional Animal Care and Use Committee at Washington University School of Medicine in St. Louis, MO (protocol numbers 20140114 and 20170081).

### Bacterial strains

The *E*. *coli* isolates used were a kanamycin-resistant derivative (UTI89 attHK022::COMGFP) of the human cystitis isolate UTI89 [19], a kanamycin resistant derivative (CFT073 HK::Kn) of the human urosepsis isolate CFT073 [20], and a spectinomycin-resistant derivative (MG1655 attP::pPSSH10) of the lab strain MG1655 [21]. Strains were cultured statically in lysogeny broth (LB) at 37°C for two consecutive overnight passages, growth conditions commonly used for uropathogenic *E*. *coli* to promote type 1 pilus expression [22].

### Mouse infections

Female, five to seven weeks old, non-pregnant C57BL/6 mice from the Jackson Laboratory were used for all experiments except the duration of the estrus phase after estrogenization, which used C57BL/6 mice from both Jackson Laboratory and Envigo. For initial experiments, mice were housed in static (non-ventilated) micro-isolator cages. At later stages of the research, our animal facility moved to a new housing system where larger cages are docked in HEPA-filtered ventilation racks that provide airflow control. In the three days prior to inoculation, mice received two doses of 0.5 mg β-estradiol 17-valerate (Sigma, St. Louis, MO, USA) in 100 μl filter-sterilized sesame oil (Fisher, NH, USA) via intraperitoneal injection to synchronize the mice in estrus [23]. At the time of infection, 10^4^ colony-forming units (CFU) of bacteria were resuspended in phosphate-buffered saline (PBS) and inoculated into the vaginas in 20 μl volumes. For mock infections (controls for histology and flow cytometry experiments), mice were estrogenized, then inoculated with 20 μl PBS and housed in cages separate from infected mice (except during transmission experiments where mock-infected mice were co-housed with infected mice). At experimental end points, animals were humanely euthanized by isoflurane inhalation followed by cervical dislocation. To enumerate bacterial CFUs, vaginal washes were collected using PBS as previously described [23], serially diluted and plated on MacConkey agar containing 25 μg/ml kanamycin or 20 μg/ml spectinomycin. At experimental end points, mice were humanely sacrificed and vaginas and uterine horns were harvested, weighed, homogenized and serially diluted in PBS, then plated on MacConkey agar containing 25 μg/ml kanamycin or 20 μg/ml spectinomycin for CFU enumeration.

### Observations of the estrous cycle

To monitor the estrous cycle when testing the duration of estradiol treatment, vaginal washes were collected after 1, 2, 3, 6, 8, 10, 13, 15, 17, 20, 22, 25, 27, 29 and 31 days (a total of 15 times over the 31-day experiment) and wet mounts were prepared and observed at 20x magnification on a Nikon (Tokyo, Japan) Diaphot inverted microscope. Images were acquired using an AmScope (CA, USA) microscope digital camera (MU300, 3.1 MP). Representative images were assessed by four independent investigators and the consensus date on which each mouse came out of estrus was plotted; at least three out of four investigators agreed for each mouse.

### Vaginal wash scoring

To assess immune cell infiltration into vaginal wash fluid, at indicated time points, 5 μl of vaginal wash was smeared onto a slide and stained with the Hema 3 staining kit (Fisher, NH, USA) after air drying. Immune cells and epithelial morphology were assessed on an Olympus (Tokyo, Japan) BX61 bright field microscope.

### Histopathology

The reproductive tract (vagina, cervix, and uterine horns) was harvested as an intact structure, fixed overnight in 10% neutral-buffered formalin (Fisher, NH, USA), paraffin-embedded and sectioned. Slides were stained with hematoxylin and eosin, then imaged with an Olympus (Tokyo, Japan) BX61 bright field microscope. An observer blinded to the infection status of the samples scored the level of polymorphonuclear cells in the tissue based on the following rubric: 0 –absent, 1 –minimal, 2 –moderate, 3 –profound, 4 –severe (including immune cells crossing the epithelium into the lumen).

### Flow cytometry

Replicates were conducted with five mice per group (1–2 mock-infected with PBS and 3–4 *E*. *coli* UTI89-infected). At 12 days post-infection, mice were humanely euthanized without perfusion and the vagina, cervix and uterine horns were collected and kept on ice in 1 ml RPMI buffer containing 1% fetal bovine serum, 1% penicillin/streptomycin, and 50 μM 2-mercaptoethanol. Single cell suspensions were generated and flow cytometry was performed as previously described [24]. The following antibodies were used for this study: TruStain fcX (BioLegend, CA, USA, clone 93), Siglec-F (BD Pharmingen, NJ, USA, clone E50-2440), LyG6 (BioLegend, clone 1A8), CD11b (BioLegend, clone M1/70), CD11c (BioLegend, clone N418), Ly6C (BioLegend, clone HK1.4), F4/80 (BioLegend, clone BM8), and I-A/I-E (BioLegend, clone M5/114.15.2). CountBright beads (Invitrogen, CA, USA) were added to samples prior to acquisition to determine cell counts.

### Statistical analysis

All relevant data, including individual data points (not just central tendency and variance measures), are presented within the paper and supplementary figures. Statistics were performed in GraphPad (CA, USA) Prism v7.01. A two-tailed Fisher’s exact test was used to test for significant differences in the incidence of uterine horn colonization and the presence of uterine horn leukocytes. Spearman’s rank correlation coefficient was used to test for significant associations among titers in vaginal washes, vaginal and uterine horn tissue, and uterine histology. For other data, comparisons of three or more groups were performed with the Kruskal-Wallis test followed by Dunn’s correction. Pairwise comparisons were performed with a two-tailed Mann-Whitney U test. *P* ≤0.05 was considered statistically significant. For histology scoring, investigators were blinded to the infection status of the animals.

## Results

**Fig 1:**
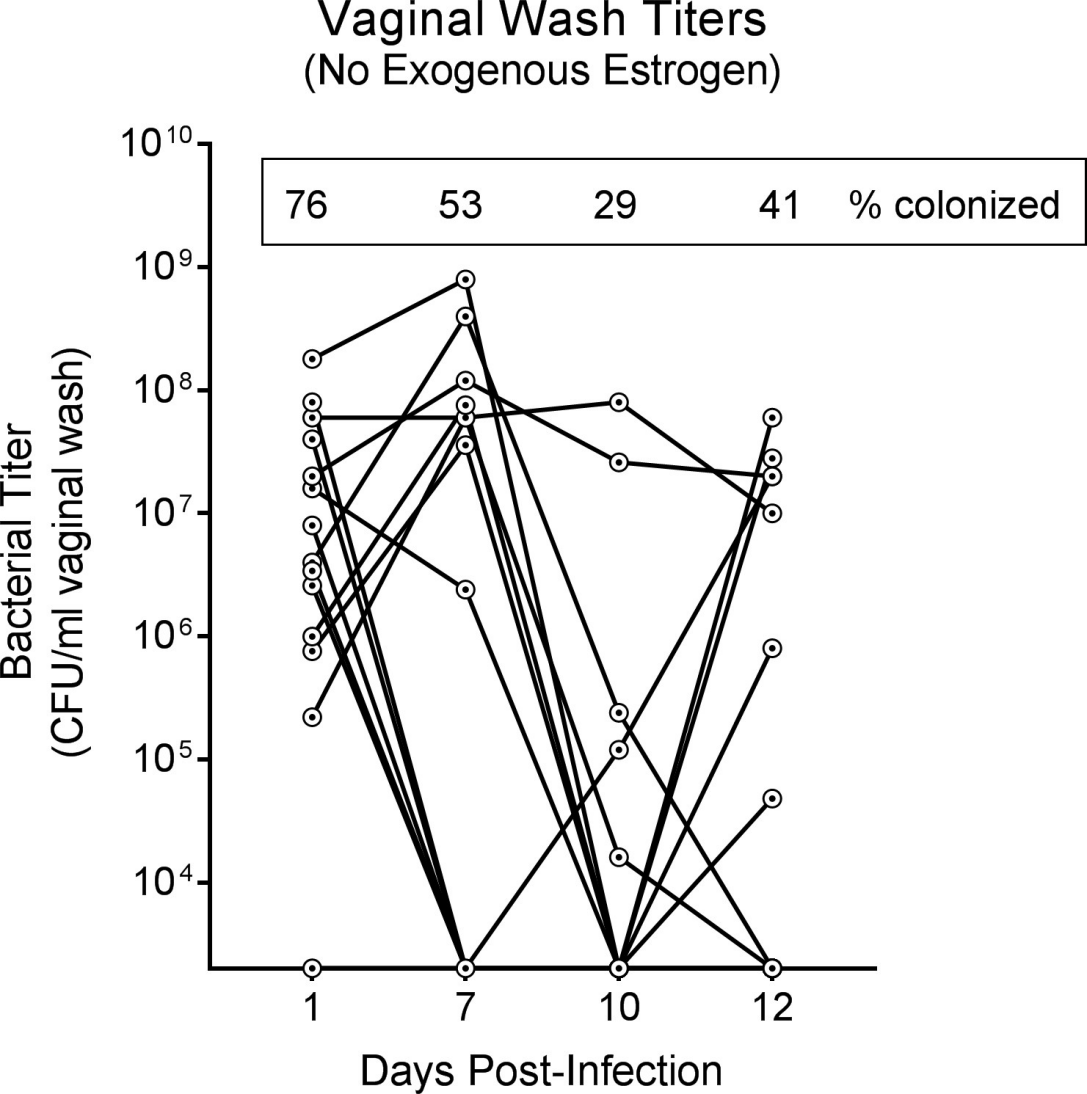
Vaginal *E*. *coli* titers wax and wane in mice not treated with exogenous estrogen. Seventeen non-pregnant female C57BL/6 Jackson mice were vaginally inoculated with *E*. *coli* strain UTI89, and vaginal washes were collected to monitor infection status over 12 days. Each point shows an individual mouse, with lines connecting the titers of a given mouse from one time point to the next. The percentage of mice with any detectable titer is shown in the box at the top of the graph. Data are combined from two independent experiments and zeros are plotted at the limit of detection. These experiments were conducted with static (non-ventilated) micro-isolator cages.

In this study, we sought to establish a simple mouse model of *E*. *coli* infection of the female reproductive tract. In C57BL/6 mice, after vaginal inoculation with 10^4^ colony-forming units (CFU) of the clinical *E*. *coli* isolate UTI89, *E*. *coli* titers in vaginal washes rose and fell over time (Fig 1 and S1 Fig). Of 17 mice tested in two replicates, two had stably high titers over time, three never had detectable titers at any time point, and 12 had variable titers that were sometimes high, sometimes low and sometimes undetectable. The percentage of mice with any detectable titer was greatest at one day post-infection (dpi), with titers detected in 14 mice (76%), and decreased over time.

**Fig 2:**
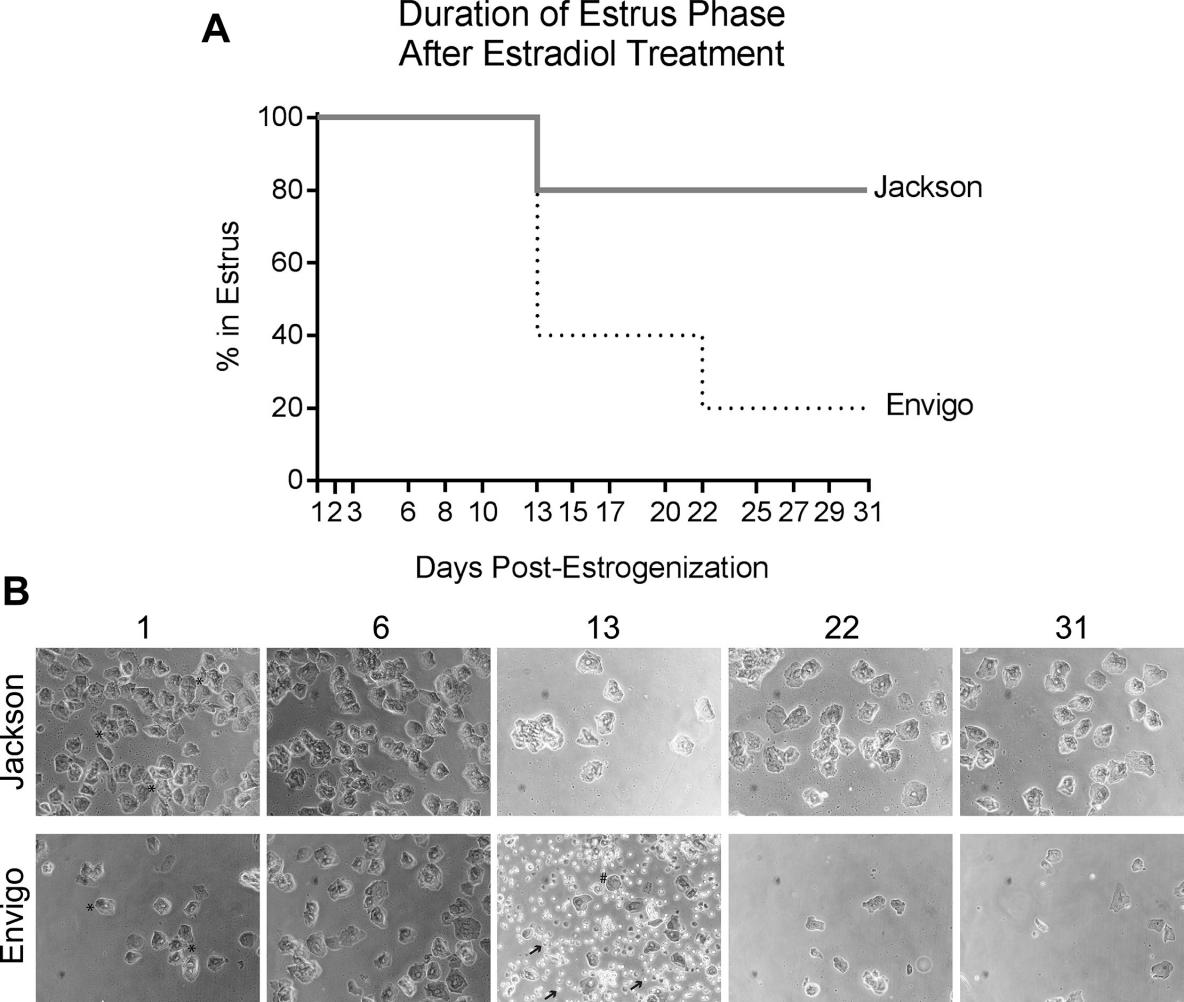
After exogenous estrogen treatment, some C57BL/6J mice stay in the estrus phase for several weeks. C57BL/6 mice from Jackson or Envigo were given two doses of β-estradiol 17-valerate 72 hours apart and followed for one month. (**A**) Vaginal (PBS) washes were collected at the indicated 15 time points over 31 days and the stage of the estrous cycle was determined by wet mount microscopy according to the criteria in [27]. Data are from one experiment with n = 5 mice per group. (**B**) Shown are wet mount images at 20x magnification, taken at the indicated days post-estrogenization, from one representative Jackson mouse that did not come out of estrus during the experiment (top row) and one representative Envigo mouse that came out of estrus on day 13 (bottom row). Estrus is defined as an abundance of cornified epithelial cells in washes (examples marked with * in the first column); as mice leave estrus these are replaced by more rounded epithelial cells (examples marked with # in the third column) and leukocytes (examples marked with arrows in the third column). These experiments were conducted with static (non-ventilated) micro-isolator cages.

**Fig 3:**
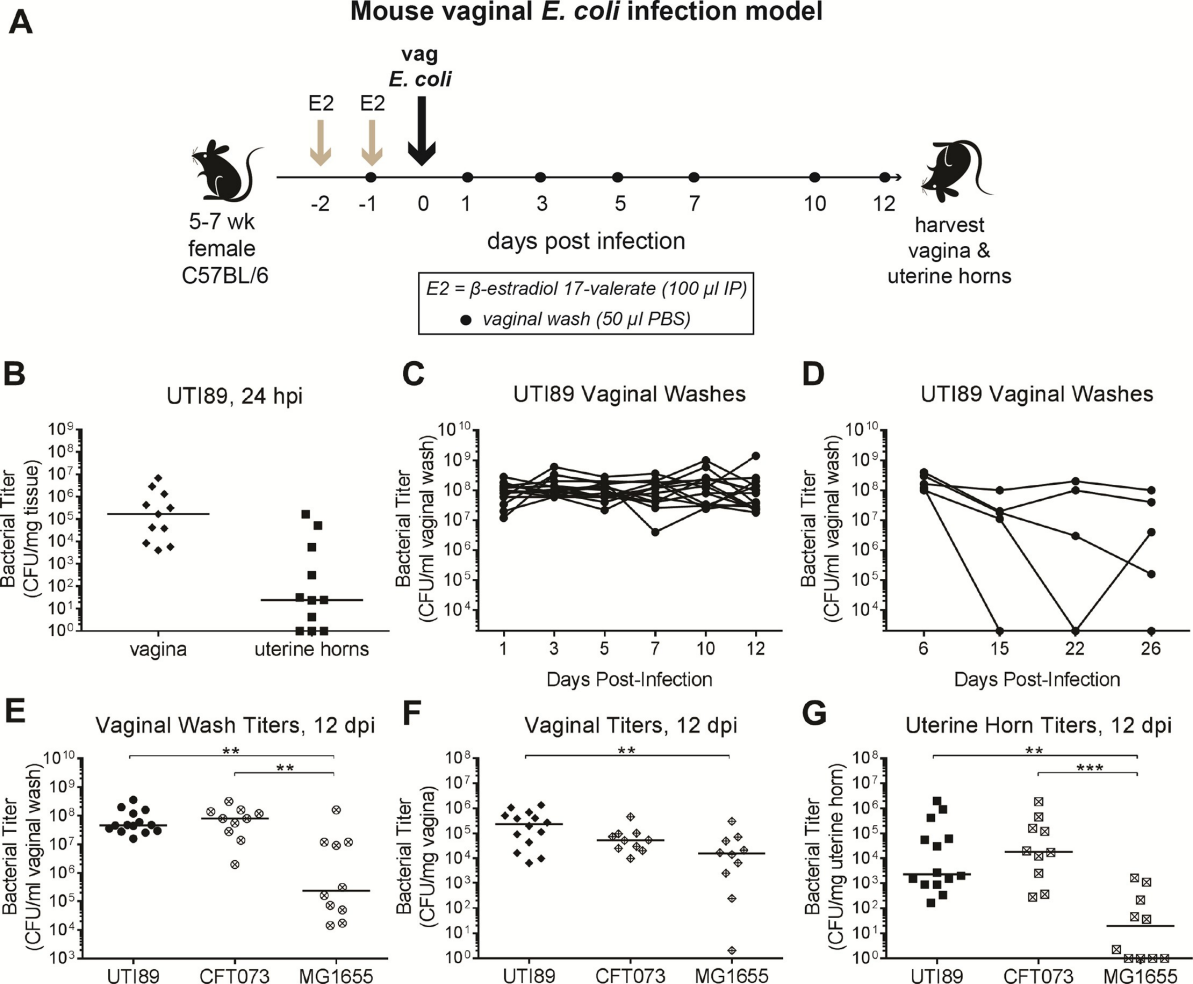
Clinical uropathogenic *E*. *coli* isolates robustly colonize the female reproductive tract of estrogenized mice. (**A**) Mouse vaginal *E*. *coli* infection model. Non-pregnant female C57BL/6 Jackson mice were given two doses of β-estradiol 17-valerate (E2) and vaginally inoculated with *E*. *coli* strains, then followed for 12 days. Vaginal washes were collected to monitor infection status. (**B through D**) Mice were infected with UTI89. Bacterial titers were enumerated in vaginal and uterine horn tissues after 24 hours (**B**) and in vaginal washes in 12 day infections (**C**) and a longer-term (26 day) infection (**D**). **(E through G**) Mice were infected with the *E*. *coli* strains UTI89 (n = 14), CFT073 (n = 10) or MG1655 (n = 10) for 12 days. Bacterial titers were enumerated in vaginal washes (**E**), vaginal homogenates (**F**) and uterine horn (UH) homogenates (**G**). Data are combined from two or more independent experiments except for (**B** and **D**); zeros are plotted at the limit of detection and bars indicate median values. ** *P* < 0.01, *** *P* < 0.001, Kruskal-Wallis test with Dunn’s correction. hpi, hours post-infection; dpi, days post-infection. These experiments were conducted with static (non-ventilated) micro-isolator cages.

We reasoned that the heterogeneity we observed in *E*. *coli* vaginal colonization in Fig 1 could be the result of mice being in different phases of the estrous cycle. Indeed, established mouse models of vaginal bacterial infection have circumvented this issue by arresting the estrous cycle with β-estradiol 17-valerate [23, 25], which keeps mice in pseudo-estrus, marked by an abundance of cornified epithelial cells, few nucleated epithelial cells, and an absence of leukocytes in vaginal washes [26]. These prior studies did not report how long β-estradiol treatment is effective, so we first assessed the duration of the estrus phase in estrogenized mice for one month after treatment. C57BL/6 mice from Jackson or Envigo were given two doses of 0.5 mg β-estradiol 17-valerate by intraperitoneal injection and vaginal washes were collected over time and scored according to the criteria in [27] (Fig 2). Interestingly, we observed a vendor effect: three of five Envigo mice exited estrus by 13 days post-treatment, and by 31 days, only one mouse was still in estrus. In contrast, only one Jackson mouse exited estrus (by day 13) during the 31-day period. Thus, for subsequent experiments we used only estrogenized C57BL/6J (Jackson) mice (Fig 3A).

We found that 24 hours after vaginal inoculation with 10^4^ CFU of UTI89, estrogenized mice had vaginal titers clustered around 10^5^ CFU/mg tissue, with a wider spread of uterine horn titers ranging from undetectable to 10^5^ CFU/mg (Fig 3B). We wondered whether colonization would be stable over time in estrogenized mice. Indeed, we detected consistently high vaginal wash titers over the course of 12 days (Fig 3C), between 10^7^ and 10^8^ CFU/ml. Some mice remained colonized for three weeks or more (Fig 3D), in keeping with the long duration of estrus in estrogenized Jackson mice.

The vagina is considered a reservoir for uropathogenic *E*. *coli*, which may ascend through the urethra to establish urinary tract infections [28]. Thus, we compared FRT infection with the well-characterized urinary tract infection clinical isolates UTI89 and CFT073 versus the lab strain MG1655, in each case using a 10^4^ inoculum. Bacterial titers were enumerated at 12 dpi (Fig 3E–3G). Both UTI89 and CFT073 colonized the FRT robustly, with median titers of ~10^8^ CFU/ml vaginal wash, ~10^5^ CFU/mg vaginal tissue, and ~10^3^ to ~10^4^ CFU/mg uterine horn tissue; despite high bacterial titers, mice did not appear sick (no hunching, hair loss, lethargy, etc.). In contrast, MG1655 titers were statistically significantly lower throughout the FRT. Strikingly, 4 of 10 mice infected with MG1655 had no recoverable uterine horn CFUs, whereas bacteria were recovered from the uterine horns of all mice infected with UTI89 (*P* < 0.05, Fisher’s exact test) or CFT073.

**Fig 4:**
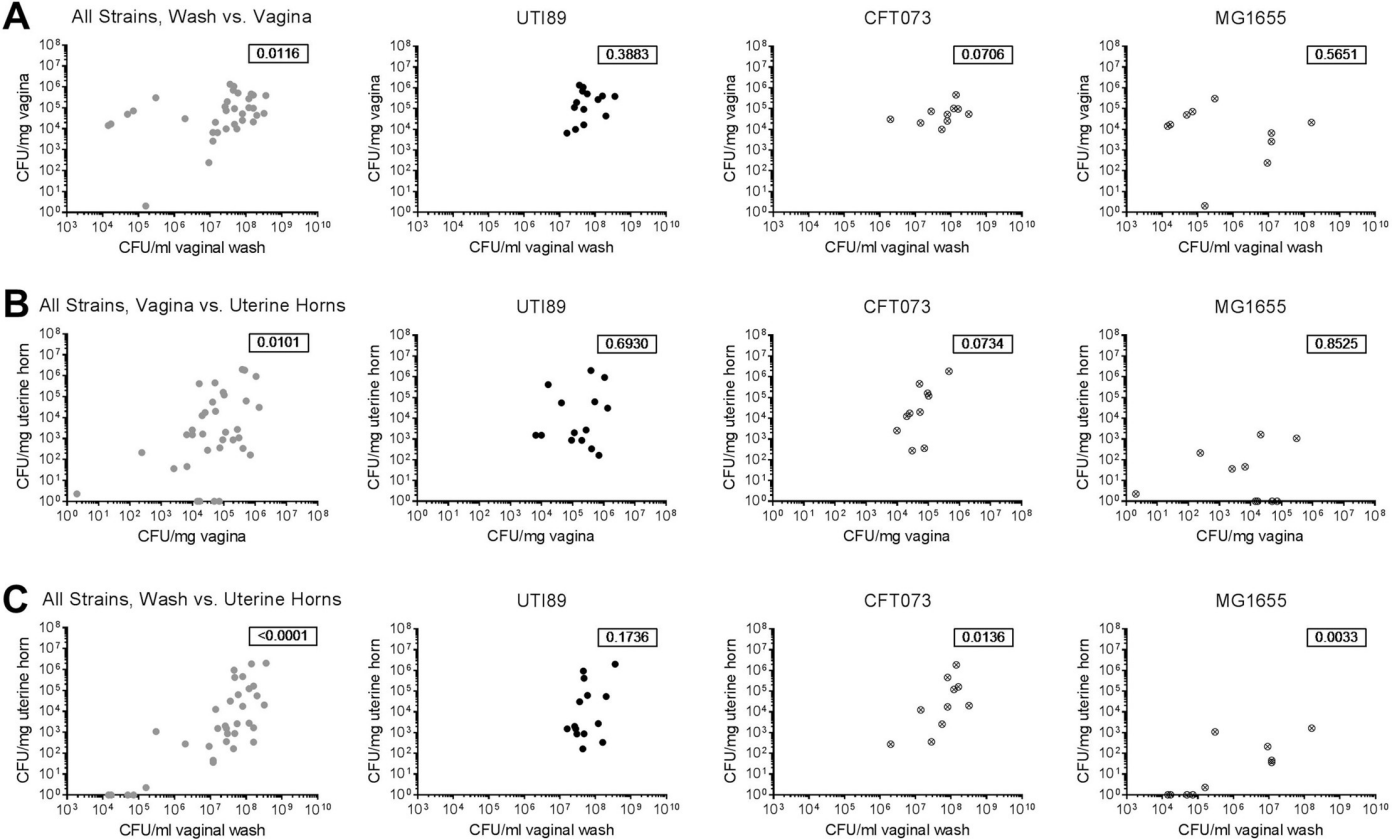
Uterine horn titers are significantly positively correlated with titers in the vagina and vaginal washes. The *E*. *coli* titers from all mice shown in **Fig 3E–3G** were assessed by Spearman correlations comparing vaginal washes vs. vaginal homogenates (**A**), vaginal homogenates vs. uterine horn homogenates (**B**), and vaginal washes vs. uterine horn homogenates (**C**). The first column shows all bacterial strains (UTI89, CFT073, and MG1655) combined, and the second, third and fourth columns show UTI89, CFT073 and MG1655 individually. The Spearman correlation *P* value is indicated in the top right of each graph. Data are combined from two or more independent experiments and zeros are plotted at the limit of detection. These data come from experiments conducted with Jackson mice housed in static (non-ventilated) micro-isolator cages.

To test whether *E*. *coli* uterine infection reflects the extent to which the vagina becomes robustly colonized, we looked for significant correlations among titers of UTI89, CFT073 and MG1655 in vaginal washes, vaginal tissue and uterine horn tissue with Spearman correlation analysis. When data for all three *E*. *coli* strains were combined, titers in the vaginal washes were significantly correlated with titers found in the vaginal homogenates (Fig 4A, first panel, *P* < 0.05), as expected. As well, titers in the uterine horn homogenates were significantly correlated with both vaginal titers (Fig 4B, first panel, *P* < 0.05) and vaginal wash titers (Fig 4C, first panel, *P* < 0.0001), suggesting that indeed, ascending uterine horn infection in this model may directly reflect the robustness of vaginal colonization. However, when we tested correlations for each strain individually, we found that UTI89 titers were not significantly correlated in any comparison (Fig 4A–4C, second panels). CFT073 titers were significantly correlated or trended toward significance for all comparisons (Fig 4A–4C, third panels), and only MG1655 vaginal wash and uterine horn titers were significantly correlated (Fig 4A–4C, fourth panels). These data suggest that bacterial strain-specific differences may impact the association between vaginal and uterine colonization in this model.

**Fig 5:**
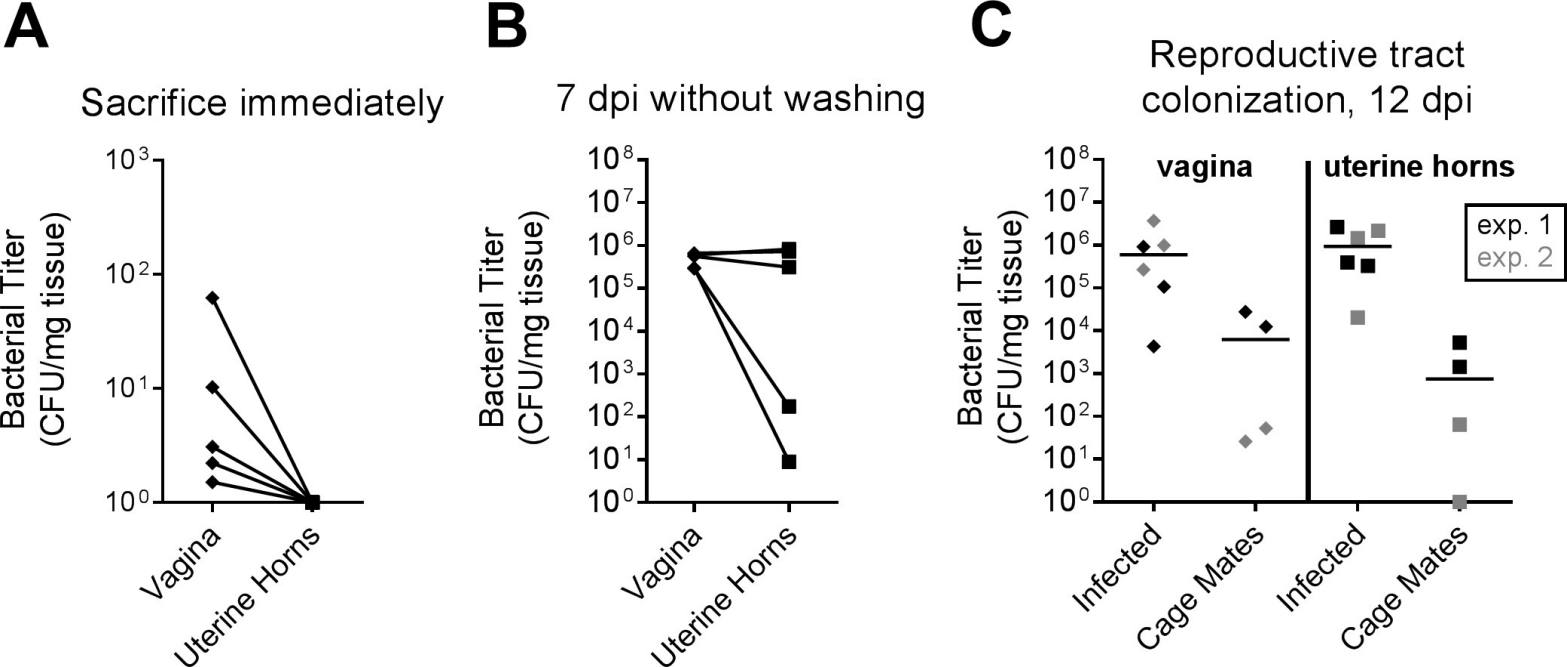
Ascension to the upper FRT is not due to techniques employed during vaginal inoculation or washing, and *E*. *coli* in the FRT can be spontaneously transmitted among estrogenized cage mates. Shown are bacterial titers in the vagina and uterine horns. (**A**) Estrogenized mice were vaginally inoculated with 10^4^ CFU of UTI89 and immediately sacrificed. (**B**) Estrogenized mice were vaginally inoculated with 10^4^ CFU of UTI89 and sacrificed at seven dpi without collecting vaginal washes. (**C**) In two experiments (the first shown in black and the second in grey), a cage of five mice were estrogenized. Two were mock-infected with PBS and three were infected with 10^4^ CFU of UTI89. At 12 dpi, UTI89 was detected in all infected mice, and was also detected in the vagina of four out of four mock-infected cage mates and in the uterine horns of three out of four mock-infected cage mates. All mice came from the Jackson Laboratory; experiments from panels **A** and **B** and replicate one in panel **C** were performed with static (non-ventilated) microisolator cages, and replicate two in panel **C** was performed with HEPA-filtered cages with airflow control. Zeros are plotted at the limit of detection and bars indicate median values.

We considered whether the acts of vaginal inoculation and/or washing may have introduced *E*. *coli* into the uterine horns. However, *E*. *coli* was not detectable in the uterine horns of mice sacrificed immediately after vaginal inoculation of 10^4^ CFU of UTI89 (Fig 5A). As well, similar levels of UTI89 were recovered from the uterine horns at seven dpi in all (four of four) mice that were never vaginally washed following inoculation (Fig 5B, compare to Fig 3G). Finally, we observed spontaneous *E*. *coli* transmission from infected to mock-infected (PBS-inoculated) cage mates. In these experiments, three UTI89-infected mice were co-housed with two mock-infected mice (all estrogenized). At 12 dpi, vaginal and uterine horn titers were enumerated. Two experiments of this kind were performed with different housing conditions (see Methods). In both experiments, kanamycin-resistant UTI89 could be recovered from both vaginal and uterine horn homogenates of co-housed, mock-infected animals (Fig 5C). Taken together these findings demonstrate that in estrogenized mice, ascending infection of the uterine horns can occur with low-level vaginal inoculation of *E*. *coli* or following transmission between cage mates, and does not require investigator intervention.

**Fig 6:**
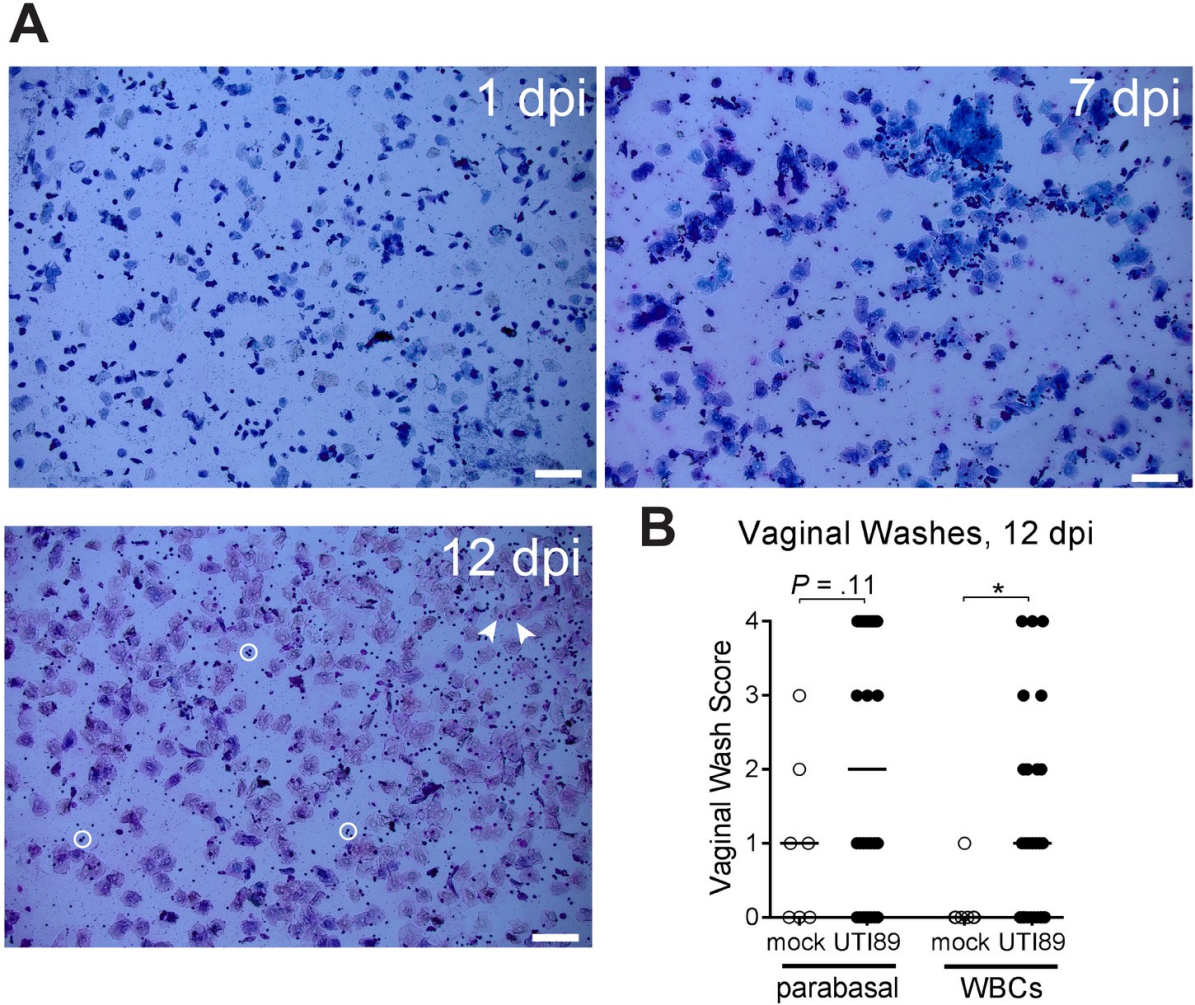
Vaginal washes from infected mice contain white blood cells. Vaginal washes from estrogenized Jackson mice were collected at the indicated time points and examined by light microscopy. (**A**) White blood cells (examples indicated with circles) and exfoliated vaginal epithelial cells (examples indicated with arrows) were observed in washes. Scale bars = 100 μm. (**B**) Vaginal washes from UTI89-infected and mock-infected (PBS-inoculated) mice were scored for the presence of parabasal cells and white blood cells (WBCs) in a blinded fashion. * *P* < 0.05, Mann-Whitney U test. These experiments were conducted with static (non-ventilated) micro-isolator cages.

During the normal estrous cycle, white blood cells infiltrate the vaginal lumen after ovulation (metestrus and diestrus); keeping mice in a pseudo-estrus phase via administration of β-estradiol 17-valerate prevents this influx of white blood cells. Here, light microscopy of Hema 3-stained smears of vaginal washes collected at one, seven and 12 days post-UTI89 infection revealed white blood cells and parabasal epithelial cells (Fig 6A). A blinded assessment revealed significantly more white blood cells in infected mice compared to mock-infected (PBS-inoculated) controls, and higher numbers of parabasal cells, though not statistically significant (*P* = 0.11) (Fig 6B).

**Fig 7:**
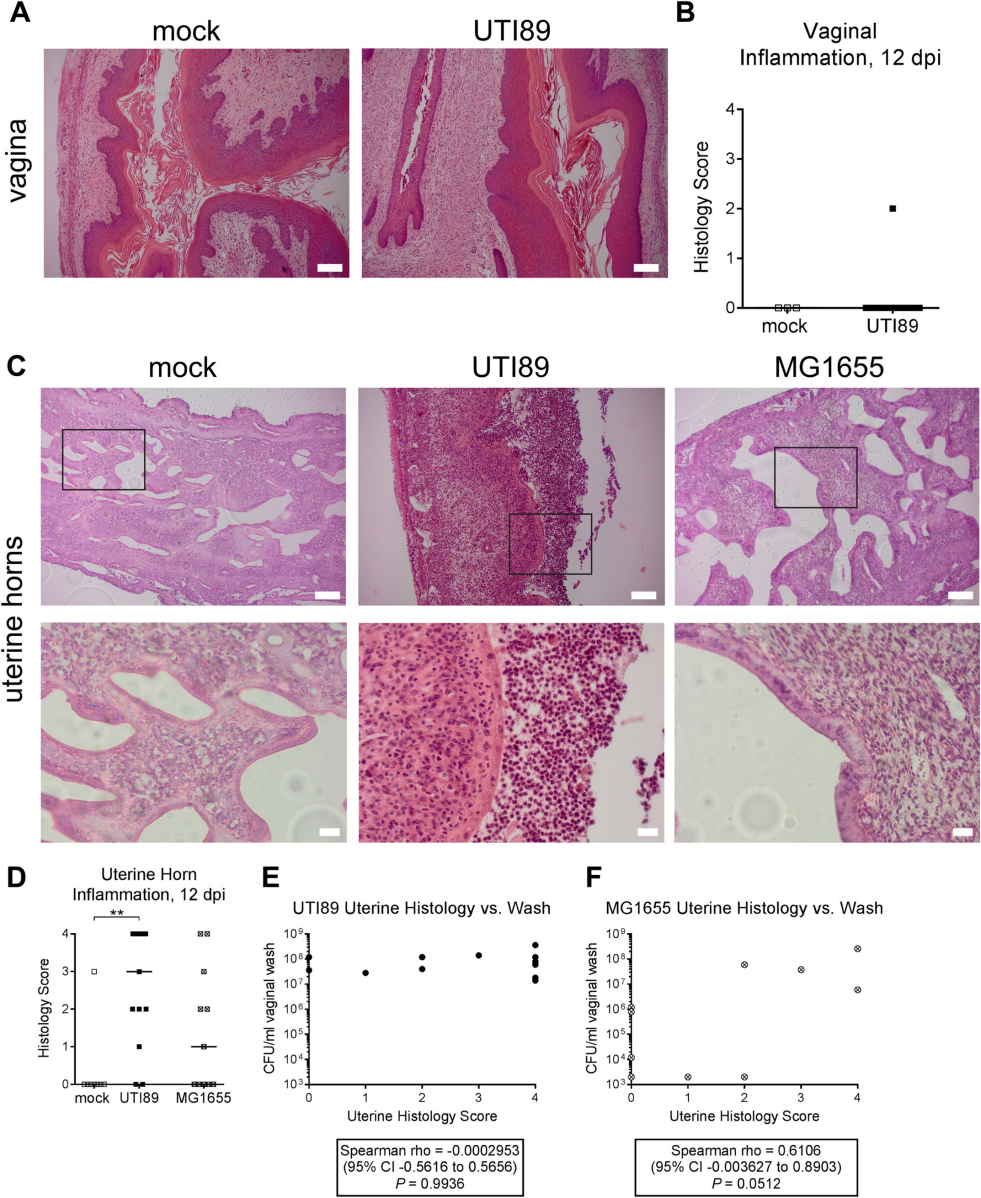
*E*. *coli* infection elicits inflammation in the uterine horns but not the vagina. Estrogenized Jackson mice were infected with the *E*. *coli* strains UTI89 or MG1655, or mock-infected with PBS. After 12 days, inflammation was assessed in vaginal and uterine horn tissues by a blinded assessment of hematoxylin and eosin-stained tissue sections. (**A**) Representative images of vaginal tissue from mock-infected vs. UTI89-infected mice. Scale bars, 100 μm. (**B**) Blinded scoring of vaginal inflammation, where 0 = absent (normal tissue with no immune infiltrate in epithelium), 1 = minimal, 2 = moderate, 3 = profound and 4 = severe (profound tissue inflammation plus immune cells crossing the epithelium into the lumen). (**C**) Representative images of uterine horn tissue from mock-infected vs. UTI89 or MG1655-infected mice. Scale bars on the top row are 100 μm; squares indicate regions shown at higher magnification on the bottom row, with scale bars indicating 20 μm. (**D**) Blinded scoring of uterine horn inflammation, using the same scoring criteria from panel **B**. ** *P* < 0.01, Kruskal-Wallis test with Dunn’s correction. (**E and F**) Spearman’s correlation was used to compare vaginal wash titers collected at the time of sacrifice to the uterine horn histology scores shown in panel **D** for mice infected with UTI89 (**E**) or MG1655 (**F**). Data are from four independent experiments conducted in both static (non-ventilated) micro-isolator cages and HEPA-filtered cages with airflow control, with similar results between conditions.

Since estrogenization suppresses vaginal leukocyte infiltration, we sought to further characterize the source of the inflammatory infiltrates in the FRT in response to *E*. *coli* infection. We first performed blinded scoring of tissue sections collected at 12 dpi from infected and mock-infected (PBS-inoculated) mice. We observed no evidence of vaginal histological inflammation (inflammatory score of zero) in three of three mock-infected mice and ten of 11 UTI89-infected mice (with one mouse having a score of two, defined by moderate infiltration of polymorphonuclear cells) (Fig 7A and 7B), suggesting that the white blood cells present in vaginal washes were not vaginal in origin. In the uterine horns, we observed polymorphonuclear leukocytes (PMNs) in the tissue of one of eight mock-infected mice, and in the tissue and/or lumen of 11 of 13 UTI89-infected mice (P < 0.01, Fisher’s exact test) (Fig 7C and 7D). PMNs were also present in the uterine horn tissue and/or lumen in six of 11 MG1655-infected mice (Fig 7C and 7D), though only two of 11 had the highest score (defined by profound tissue inflammation plus immune cells crossing the epithelium into the lumen) compared to six of 13 UTI89-infected mice. Comparable levels of inflammation were seen in each horn of a given mouse. Because both horns were used for histology, bacterial titers of the uterine horns are not known for these experiments; however, vaginal washes were collected. UTI89 vaginal wash titers were not significantly correlated with uterine horn histology scores (Fig 7E); although there was a wide spread of uterine histology scores, UTI89 vaginal wash titers were all clustered between 10^7^ and 10^8^ CFU/ml vaginal wash. In contrast, MG1655 vaginal wash titers and uterine histology scores suggest a different relationship in which higher bacterial numbers were seen alongside higher uterine inflammatory scores, but a positive correlation did not quite reach statistical significance (*P* = 0.0512) (Fig 7F).

**Fig 8:**
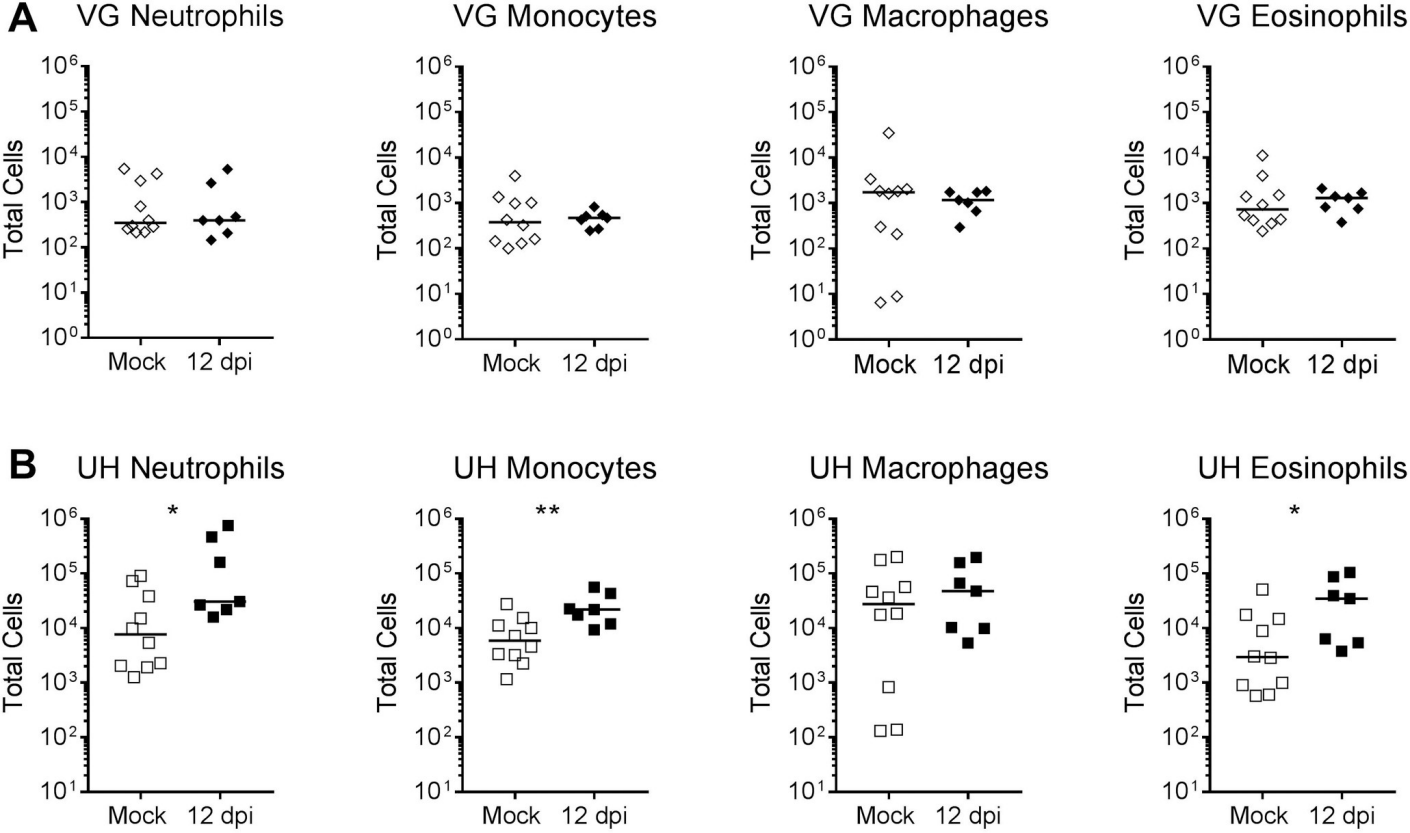
Flow cytometry demonstrates increased neutrophil, monocyte and eosinophil infiltration into the uterine horns upon *E*. *coli* infection. Flow cytometry was performed on vaginal (VG, panel **A**) and uterine horn (UH, panel **B**) single cell suspensions from mock-infected (PBS-inoculated) or UTI89-infected Jackson mice. The gating strategy used was: Siglec-F^-^, Ly6G^+^ cells were considered neutrophils; Siglec-F^-^, Ly6G^-^, CD11b^+^, CD11c^-^, Ly6C^+^ cells were considered monocytes; Siglec-F^-^, Ly6G^-^, CD11b^+^, CD11c^-^, Ly6C^-^, F4/80^+^ cells were considered macrophages; and Siglec-F^+^ cells were considered eosinophils. * *P* < 0.05, ** *P* < 0.01, Mann-Whitney U test. Data are combined from two or more independent experiments; data points represent actual values for each individual mouse and bars indicate median values. These experiments were conducted with static (non-ventilated) micro-isolator cages.

To further characterize immune cell infiltration into the infected FRT, we performed flow cytometry to enumerate neutrophils, monocytes, macrophages, and eosinophils at 12 dpi in UTI89-infected mice relative to mock-infected (PBS-inoculated) mice (see S2 Fig for our gating strategy). In accordance with tissue histology, we did not observe higher levels of any of the above cell types in infected vaginal tissue compared to the mock-infected control group (Fig 8A). However, infected uterine tissues had significantly higher numbers of neutrophils, monocytes and eosinophils compared to mock-infected uterine horns (Fig 8B). Evaluation of these immune cells in cervical tissue did not reveal differences in the numbers of these immune cell subsets between mock-infected and *E*. *coli*-infected animals, suggesting that the cervix is not the source of these immune cells (S3 Fig). Thus, ascending *E*. *coli* infection of the FRT elicits a strong uterine inflammatory response and severe immunopathology in the uterine horns, despite potential immunosuppressive effects of exogenous estrogen treatment.

## Discussion

*E*. *coli* is a relatively common pathogen of the FRT, and ascending *E*. *coli* infection causes a variety of negative health outcomes [13, 29]. Here we describe a model of ascending *E*. *coli* infection in non-pregnant mice. Vaginal inoculation with low doses of the clinical uropathogenic *E*. *coli* isolates UTI89 and CFT073 resulted in robust colonization of the FRT over 12 days, while titers of the lab strain MG1655 were significantly lower, especially in the uterine horns. UTI89 infection elicited leukocyte accumulation in vaginal washes, as well as significant uterine inflammation observed by histological analysis. Flow cytometry revealed significantly more neutrophils, monocytes and eosinophils in infected vs. mock-infected uterine horns. Thus, ascending *E*. *coli* FRT infection and resulting uterine inflammation can be modeled in mice without surgical inoculation.

It is important to note that most of the experiments presented here employed treatment with exogenous estrogen (so-called “estrogenization”) to keep mice in pseudo-estrus and prevent normal cycling. Although most non-estrogenized mice did exhibit *E*. *coli* titers in vaginal washes to varying extents, consistently high titers only occurred in estrogenized mice. Many mouse models of vaginal infection require the arrest of the estrous cycle for robust infection: either with medroxyprogesterone acetate (Depo-Provera) to keep mice in diestrus for viral infections [30, 31], or by estrogenization with β-estradiol 17-valerate (E2) to keep mice in estrus for bacterial infections [23, 25]. The estrus phase comprises abundant cornified epithelial cells and an absence of leukocytes in vaginal washes [26], conditions which may favor *E*. *coli* colonization. Estrogenization prevents the normal influx of leukocytes into the vagina that occurs during diestrus, and has other immunosuppressive effects [32]. The fluctuating vaginal *E*. *coli* levels that we observed in non-estrogenized mice may be due to normal leukocyte infiltration during the estrous cycle. As well, the apparent lack of vaginal inflammation in estrogenized mice at 12 dpi, despite sustained, robust *E*. *coli* colonization, may be attributable to local immunosuppression due to estrogenization. Nonetheless, we observed abundant leukocytes in vaginal washes from infected, estrogenized mice as early as one dpi. Tissue histology and flow cytometry at 12 dpi suggested that the uterine horns, not the vagina, were the source of the inflammatory cells. Thus, the immunosuppressive effects of estrogen can be overcome, at least in the uterine horns. We note that uterine inflammation does not appear to be a general consequence of Gram-negative bacterial infection in estrogenized mice, as high-titer *Prevotella bivia* ascending infection resulted in neither localized (uterine) nor systemic inflammation by histological analysis and/or flow cytometry at two and seven dpi [33]. Our finding of uterine inflammation upon *E*. *coli* infection in mice is consistent with a previous report wherein surgical infusion of *E*. *coli* directly into the uteri of non-pregnant C57BL/6 mice caused severe inflammation [34], which was attributed to TLR4-driven signaling in both epithelial and stromal cells [35]. Our non-surgical model serves as a straightforward means of testing candidate virulence factors or vaccine antigens in the context of severe innate immune cell infiltration, which could yield findings relevant to *E*. *coli* infections at other body sites, such as the gut, bladder, and kidneys–or in the neonatal lung, blood and brain.

Our finding that MG1655 titers in the FRT were statistically significantly lower than titers of the uropathogenic strains UTI89 and CFT073 echoes findings in pregnant mice that a pathogenic *E*. *coli* strain caused more robust ascending uterine infection than the lab strain MG1655 and caused significantly greater upregulation of the neutrophil chemoattractants *Cxcl1* and *Cxcl2* in uterine tissue, although in that study titers were not determined [17]. Here we observed that for the two robust colonizers, UTI89 and CFT073, *E*. *coli* titers in vaginal washes and vaginal tissue (at 12 dpi) were relatively tightly clustered around ~10^8^ CFU/ml vaginal wash and ~10^5^ CFU/mg vaginal tissue, respectively. In contrast, uterine horn titers had a much wider range of ~10^2^ to ~10^6^ CFU/mg tissue. For both CFT073 and MG1655, uterine horn titers were significantly correlated with vaginal wash titers, and for MG1655, uterine horn histology scores were also significantly correlated with vaginal wash titers, suggesting that for these two strains, the ascending infection was a direct result of robustness of the vaginal infection. UTI89 did not display significant correlations between vaginal and uterine horn titers or uterine horn histology scores, and therefore it remains unknown why UTI89 uterine infection was so robust in some animals and more muted in others. Because our histology and flow cytometry experiments used the entire organ, the uterine horn titers are not known for these experiments and cannot be directly tested for correlations with immune cell infiltrates. Future studies should test whether the degree of uterine inflammation is correlated (positively or negatively) with UTI89 uterine horn titers, to better understand the pathophysiology of ascending *E*. *coli* uterine infection.

Although UTI89 and CFT073 are both uropathogenic *E*. *coli* strains belonging to the B2 clade, and are more similar to one another than they are to MG1655, they are genetically distinct strains and some key virulence factors are only found in UTI89 [36]; for example, UTI89 encodes the cytotoxic necrotizing factor CNF1, which is not found in CFT073 or MG1655 and which could therefore impact UTI89 colonization specifically. Others have reported heterogeneity in the virulence factor profiles of *E*. *coli* isolated from the FRT of pregnant and non-pregnant women [2, 37, 38]. However, little is known about the specific bacterial factors that may mediate colonization. We propose our model as a straightforward means of testing candidate antigens for their role in FRT infection and inflammation.

A few studies examining the beneficial role of *Lactobacillus* species as vaginal probiotics have tested the ability of *Lactobacillus* to prevent or clear vaginal *E*. *coli* infection in mice [39, 40] and cynomolgus monkeys [41]. Our study highlights the importance of using multiple *E*. *coli* strains for such experiments, as we showed that strains differ in their ability to colonize the vagina. We note that mice harbor a vaginal microbiome that is distinct from the *Lactobacillus*-dominated “healthy” microbiome seen in women [42]. In the present study, we did not assess whether or how the vaginal microbiota may have changed in response to estrogenization or *E*. *coli* infection, and we did not look for any bacteria other than *E*. *coli* in the uterine horns. Thus, whether and to what extent *E*. *coli* infection may disrupt existing microbial communities–or conversely, how the composition of the endogenous microbiome may influence susceptibility to ascending infection–remain open questions.

Mouse models of *E*. *coli* bladder infection typically use a 10^7^ or 10^8^ CFU dose (for example, [22]), while here we found that a vaginal dose of 10^4^ CFU was sufficient to colonize all estrogenized animals. The observation that infected mice could apparently transmit *E*. *coli* to co-housed, uninfected mice provides further support for our conclusion that *E*. *coli* can very readily colonize the estrogenized FRT. Here we note that the second replicate of this experiment was performed after our mouse facility changed to a new housing system (see Methods). Although the overall conclusion of these experiments, that *E*. *coli* can be transferred between cage mates, held true, we did note lower overall levels of *E*. *coli* in uninfected cage mates following the change in housing. Whether these differences are due to aspects of the inoculation, such as trauma, fluid flow, anesthesia etc., and/or factors relating to the housing, such as bedding that is more dry and harboring fewer bacteria, remains to be elucidated.

Together with our observations that uropathogenic *E*. *coli* was an especially capable vaginal colonizer and that even non-estrogenized animals can carry *E*. *coli* vaginally for long periods, these findings suggest that urinary tract infection models may also result in significant *E*. *coli* FRT reservoirs, a phenomenon that may vary in intensity depending on housing conditions and other factors. There are multiple reported mouse models of chronic and/or recurrent bladder infection reported in the literature (for example, [22, 43]). The likely outcome that *E*. *coli* in urine could result in vaginal colonization and ascending uterine infection in these models requires new consideration that an FRT reservoir may contribute to certain aspects of bladder pathophysiology in mice, as it does in humans. Importantly, elegant experiments previously showed that experimental bladder infection with UTI89 in pregnant mice caused intrauterine growth restriction (low gestational and birth weights), due to inflammation-mediated “cross-talk” between organ systems [44]. Whether cross-talk may be bi-directional–that is to say, whether uterine inflammation caused by ascending uterine *E*. *coli* infection could also affect UTI outcomes–is an open question. Another potential technical consideration is that “clean catch” procedures for urine collection do not exist for mouse experiments and thus vaginal colonization could potentially contribute to *E*. *coli* titers detected in urine.

A mouse surgical infection model was previously used to study the *E*. *coli* factors necessary for uterine colonization [34, 35]. Our work shows that uterine *E*. *coli* infection can be studied without surgery, making future studies less technically challenging. As well, given the importance of *E*. *coli* uterine infection in causing pregnancy complications, a few studies have investigated vaginal *E*. *coli* inoculation in pregnant rodents [16, 17, 45], though without assessing bacterial titers, histology or immune cell infiltration. Performing vaginal infections in the context of pregnancy is essential, but also technically demanding and resource-intensive. Our model provides a less technically complicated, less invasive, and more natural means to investigate host-pathogen interactions in ascending *E*. *coli* uterine infections. We hope that this model may be used to discover bacterial virulence factors promoting ascension and inflammation, and to test therapeutic interventions. Those discoveries can then be validated in pregnancy models, which may lead to improved health outcomes.

## Supporting information

S1 Fig Vaginal *E*. *coli* titers wax and wane in mice not treated with exogenous estrogen. Seventeen non-pregnant female Jackson C57BL/6 mice were vaginally inoculated with *E*. *coli* strain UTI89, and vaginal washes were collected to monitor infection status over 12 days. Each panel corresponds to one replicate of the experiment. Each point shows an individual mouse, with lines connecting the titers of a given mouse from one time point to the next. The percentage of mice with any detectable titer is shown in the box at the top of the graph. Zeros are plotted at the limit of detection. These experiments were conducted with static (non-ventilated) micro-isolator cages.
(TIF)

S2 Fig Gating strategy used for flow cytometry experiments. Shown is a representative UTI89-infected mouse at 12 dpi. These experiments were conducted with Jackson mice housed in static (non-ventilated) micro-isolator cages.
(TIF)

S3 Fig Flow cytometry did not show significantly elevated immune populations in the cervix upon *E*. *coli* infection. Flow cytometry was performed on cervical single cell suspensions from mock-infected (PBS-inoculated) or UTI89-infected Jackson mice. The gating strategy used was: Siglec-F^-^, Ly6G^+^ cells were considered neutrophils; Siglec-F^-^, Ly6G^-^, CD11b^+^, CD11c^-^, Ly6C^+^ cells were considered monocytes; Siglec-F^-^, Ly6G^-^, CD11b^+^, CD11c^-^, Ly6C^-^, F4/80^+^ cells were considered macrophages; and Siglec-F^+^ cells were considered eosinophils. No cell populations were statistically significantly different between groups. Data are combined from two or more independent experiments; data points represent actual values for each individual mouse and bars indicate median values. These experiments were conducted with static (non-ventilated) micro-isolator cages. CX, cervix.
(TIF)
