# Supplementary figures and images for: Low-dose inoculation of Escherichia coli achieves robust vaginal colonization and results in ascending infection accompanied by severe uterine inflammation in mice
Source: PLoS One. 2019 Jul 22;14(7):e0219941. doi: 10.1371/journal.pone.0219941 (PMC6645506; doi:10.1371/journal.pone.0219941)

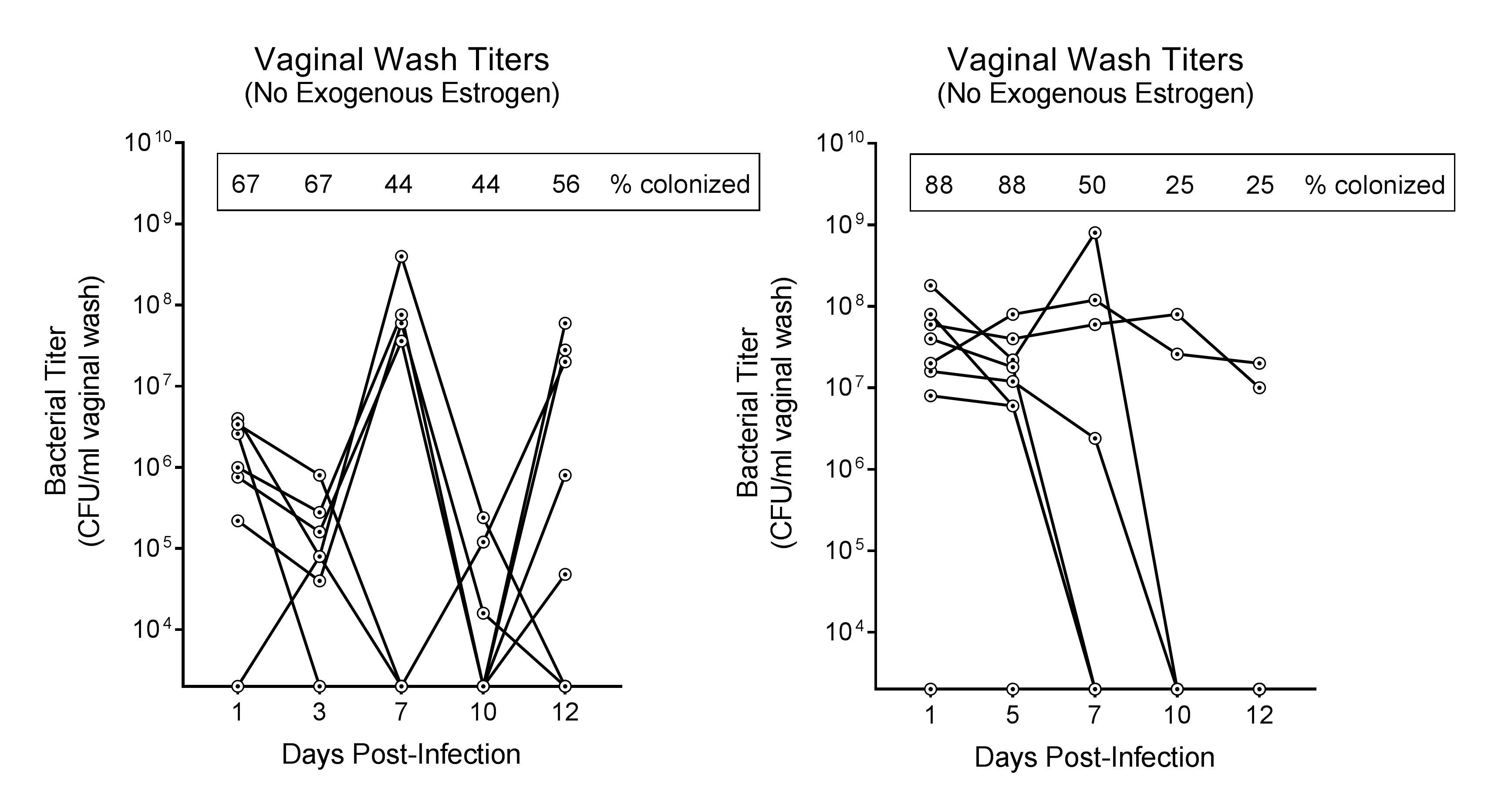

Supplement: S1 Fig — Seventeen non-pregnant female Jackson C57BL/6 mice were vaginally inoculated with E. coli strain UTI89, and vaginal washes were collected to monitor infection status over 12 days. Each panel corresponds to one replicate of the experiment. Each point shows an individual mouse, with lines connecting the titers of a given mouse from one time point to the next. The percentage of mice with any detectable titer is shown in the box at the top of the graph. Zeros are plotted at the limit of detection. These experiments were conducted with static (non-ventilated) micro-isolator cages. (TIF) [file pone.0219941.s001.tif]

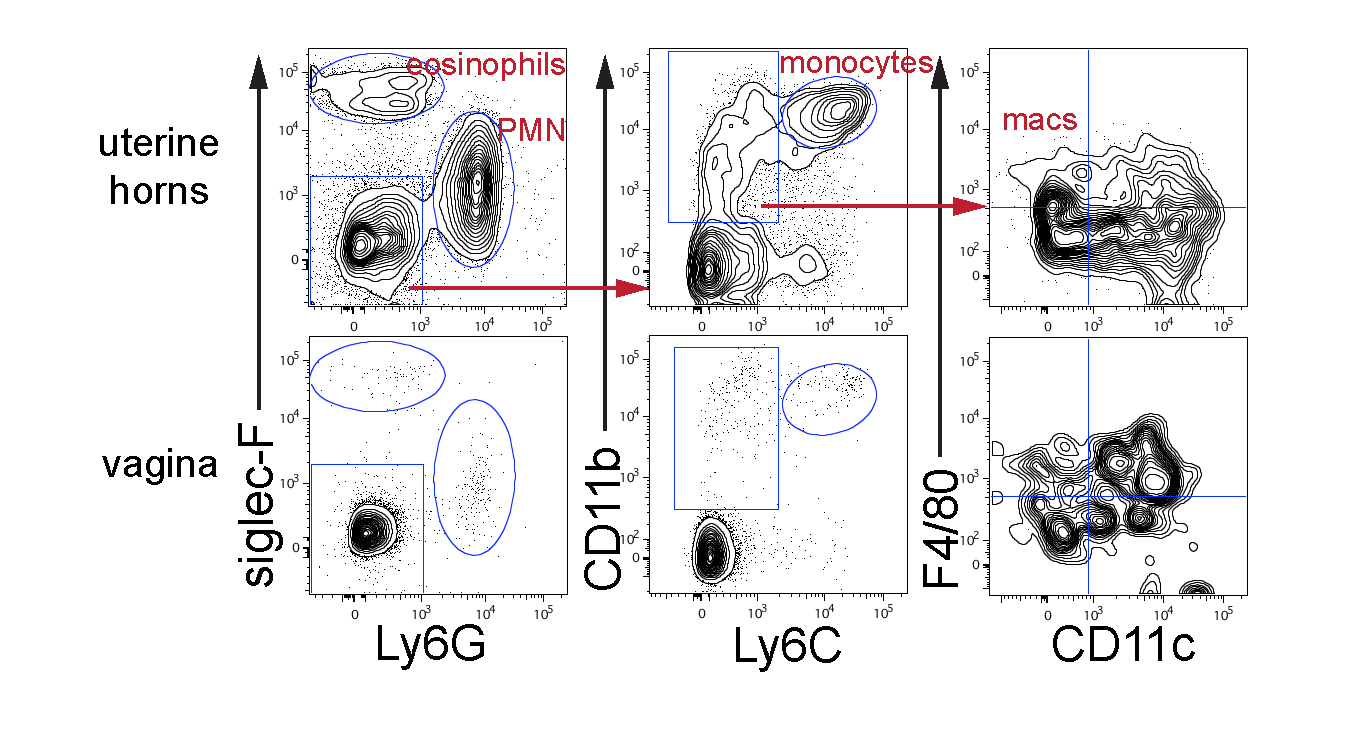

Supplement: S2 Fig — Shown is a representative UTI89-infected mouse at 12 dpi. These experiments were conducted with Jackson mice housed in static (non-ventilated) micro-isolator cages. (TIF) [file pone.0219941.s002.tif]

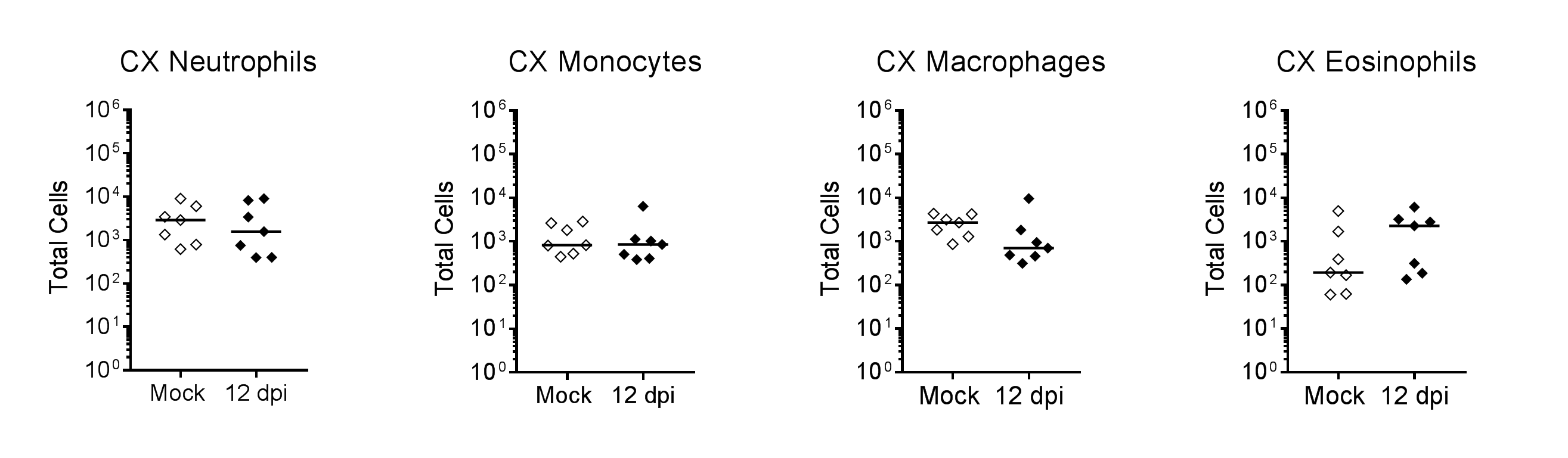

Supplement: S3 Fig — Flow cytometry was performed on cervical single cell suspensions from mock-infected (PBS-inoculated) or UTI89-infected Jackson mice. The gating strategy used was: Siglec-F-, Ly6G+ cells were considered neutrophils; Siglec-F-, Ly6G-, CD11b+, CD11c-, Ly6C+ cells were considered monocytes; Siglec-F-, Ly6G-, CD11b+, CD11c-, Ly6C-, F4/80+ cells were considered macrophages; and Siglec-F+ cells were considered eosinophils. No cell populations were statistically significantly different between groups. Data are combined from two or more independent experiments; data points represent actual values for each individual mouse and bars indicate median values. These experiments were conducted with static (non-ventilated) micro-isolator cages. CX, cervix. (TIF) [file pone.0219941.s003.tif]
